# Chicago Gynecological Society—June 19th

**Published:** 1885-08

**Authors:** 

**Affiliations:** 2330 Indiana Ave.


### SOCIETY REPORTS.

---

TRANSACTIONS OF THE CHICAGO GYNECOLOGICAL SOCIETY.

*Regular Meeting, Friday Evening, 19th June, 1885.* THE PRESIDENT, DR. H. P. MERRIMAN, in the chair.

I. ETHERIDGE. A Report of a Case of a Fœtus, Enclosed In Its Sister's Placenta. *Fœtus Compressus. Fœtus Papyraceus.*

II. BYFORD. A Report of a Case of Leio-myom of the Vagina and Uterus.

I. PROFESSOR J. H. ETHERIDGE read A REPORT OF A CASE OF A FŒTUS ENCLOSED IN ITS SISTER'S PLACENTA. (*Fœtus Compressus. Fœtus Papyraceus.*) With exhibition of the specimen.

On 26th September, 1882, Mrs. T. J. B., 22 years old, of a nervous sanguine temperament, healthy, was delivered of a mature female child, after a normal labour of four hours duration.

During the delivery of the placenta, some abnormity was detectable, which proved to be a *fœtus papyraceus.*

THE OUTER SURFACE OF THE PLACENTA.

The outer surface of the placenta at once arrests attention. A deep furrow separates the two placentæ, which are united, on their amniotic surface, by a series of compact white bands, discoverable only by pressing through the furrow. The large placenta constitutes about two-thirds of the entire mass. The smaller placenta is thin, flat and compact, being about one-third as thick as the larger one.

The placenta of the living child is normal throughout its extent. Cotyledons are well marked, the tufts and villi presenting normal microscopical characters. The placenta of the *fœtus compressus,* in about nine-tenths of its extent, is whitish-yellow, and very firm. The whole thickness of this portion of the placenta, excepting its amniotic surface, presents one unbroken mass of fatty degeneration. The remaining one-tenth of the placenta presents a carneous appearance, evidently a transition stage between normal placenta and complete fatty destruction. Its cotyledons are enmassed and its tufts and villi solidified and the whole is interspersed with initial fatty depositions.

THE FŒTAL SURFACE OF PLACENTA.

The two segments were wholly different at time of birth. The placenta of the living child presented a normal appearance. The placenta of the *fœtus papyraceus* presented the appearance of a closed bladder, which, upon examination, was found to be an unruptured amnion, containing amniotic fluid and a fœtus. The development of the *fœtus compressus* corresponded to the third month. The cord of the *fœtus papyraceus* was ten cm. longer than that of its fellow, and much thinner. The cord was inserted into the margin of the placenta, near the fully developed organ.

PATHOLOGY.

Among the causes, producing the death ot the fœtus, the following may be mentioned:

1. Faulty insertion of the cord, at the margin of the placenta, adjoining its fellow. (Kieselhausen).

2. Faulty structure of the cord; thin, twisted, or deficient in the jelly of Wharton. (C. Braun).

3. Disease of the placenta.

4. Traumatism.

5. The implanting of the umbilical vessels too closely together, and arterial anastomosis.

LITERATURE.

*Fœtus papyraceus* is of seldom occurrence. A search through the library of the Surgeon General's office at Washington resulted in finding only five references to reports of similar cases.

DISCUSSION.

DR. PHILIP ADOLPHUS thought that such cases were of more frequent occurrence than the remarks of the author of the paper would lead one to believe. In twin pregnancy, the death of one fœtus before parturition was not infrequent.

PROFESSOR W. W. JAGGARD agreed with Dr. Adolphus that while such cases were rare, a more extended research into the literature of the subject would have revealed a much larger number of cases.

While it was true that American and English text-books usually merely mentioned the fact of occurrence, German, French and Italian treatises devote a chapter to the subject. The last edition of SCHROEDER contained an excellent *résumé* of the literature. The case, reported and exhibited by Professor Etheridge, resembled in many points the case in the Pathological Museum of the Jena Lying-in Hospital, fully described by B. S. SCHULTZE. This specimen showed the placenta of a mature fœtus, and adjoining it a second egg, corresponding to the sixth week of pregnancy, with its own decidua and unruptured ammicn.

Professor Etheridge's case was chiefly interesting, as bearing upon a subject of theoretical importance, *i. e.*, superfecundation, and superfœtation.

On *á priori* grounds, it was possible that superfœtation could occur as late as the twelfth week of pregnancy,—when *decidua vera* and *reflexa* became united. Up to this time, it was possible that egg and spermatozoid might come in contact.

Superfœtation was also possible in cases of double uteri. Up to the present time, however, no case has been recorded which does not admit of a simpler explanation.

There exists a great weight of evidence in favor of superfecundation. Mares give birth simultaneously to horse and mule foals; bitches, running during the period of rut with different breeds of dogs, throw young of different, so-called bastard forms, corresponding to the breeds of the male progenitors; the same is true of cats.

A woman may give birth to twins, one of which is white, one black.

The latter fact, however, does not necessarily demand for its explanation intercourse at or near the same time with a white and a black man, since in crossing races, the offspring may resemble either father or mother, or one child may resemble the male, the other the female progenitor.

There could be no reasonable doubt as to the accuracy of Professor Etheridge's diagnosis.

DR. JOHN BARTLETT had seen one case of *fœtus compressus*, in the CHICAGO WOMAN'S HOSPITAL, about four years ago. One fœtus was mature, the other corresponded in development to the fifth month of pregnancy.

DR. BARTLETT referred to the contribution of SMELLIE and MAURICEAU upon the subject.

DR. EDWARD WARREN SAWYER referred to the fact that in ectopic pregnancy no compression of the fœtus was observed. He alluded to a case, in which he performed laparotomy three and one half years ago. The fœtus weighed eight pounds.

There could be no question about superfœtation in Professor Etheridge's case.

Fœtation, by inclusion, might be considered as explanatory of many of the monstrosities which are so commonly seen.

PROFESSOR DANIEL T. NELSON thought it would be interesting to know how much force was necessary to compress the fœtus as in Professor Etheridge's specimen. He referred to the experiments of Professor Park, of the Massachusetts Agricultural College, in the determination of the expansile force of growing squashes and pumpkins.

THE PRESIDENT wished to know whether the death of the fœtus occurred before compression, or whether it resulted from that factor. The marginal insertion of the cord doubtless was an important etiological agent. When the uterus was in the pelvic cavity, compression was greater.

He referred to the fact that in twin pregnancies, it was unusual to find both children equally developed, and frequently the birth of one preceded that of the other by minutes, hours and even days.

II. DR. HENRY T. BYFORD read A REPORT OF A CASE OF LEIO-MYOM OF THE VAGINA AND UTERUS.

The patient was a widow, about thirty-five years old. She had been married ten years, without becoming pregnant.

She had no decided symptoms of disease except an occasional back-ache, some leucorrhœa. She was treated for uterine inflammation three years before, and no tumour was discovered. Since that time, she has menstruated every two weeks. Catamenia usually lasted five days, and were normal as to quantity.

Twelve years ago, she noticed a tumour about the size of a hickory nut just within the vagina. This swelling had since that time grown steadily, until its protrusion from the vagina, about 12th of Jan., 1885, caused great inconvenience. Even at that time, the pain was promptly relieved by an opiate, administered by Dr. Doering. Two or three days subsequently, the tumor became black, swollen, and emitted a gangrenous odor. Slight septicæmia followed cauterization with nitric and carbolic acids. The tumour was attached to the anterior wall of the vagina.

The tumour was subsequently crushed off, and the patient recovered. Indagation revealed a protuberance, about the size of a goose egg, upon the right anterior surface of the uterus, which was apparently a leio-myom.

The following points of interest are to be noted in connection with the case:

1. The occurrence of both tumours in the same person.

2. The slow growth of the vaginal as compared with the uterine tumour.

3. Sloughing of the capsule, immediately after protrusion from the vagina, without impairment of the vitality of the tumour, proper.

4. Entire absence of sensitiveness to strong acids.

5. The production of a pedicle by ligaturing the proximal end with a fine wire.

6. Sterility, antedating the discovery of the uterine tumour.

7. The influence of ergot upon the uterine tumour.

DISCUSSION.

DR. EDWARD WARREN SAWYER thought the locality of the vaginal tumour was interesting but not unusual.

PROFESSOR DANIEL T. NELSON inquired whether or no fibroid tumours occurred by preference in the anterior vaginal wall.

He thought, as regards the operation, an elliptical incision around the base, and enucleation of the tumour, would have fulfilled the indications equally well.

The Society then adjourned.

W. W. JAGGARD, M. D.,
*Editor.*

2330 Indiana Ave., 24th July, 1885.

